# The burden and trends of Hepatitis B virus at a government hospital in Ghana

**DOI:** 10.4314/gmj.v59i2.3

**Published:** 2025-06

**Authors:** John G Deku, Enoch Aninagyei, Godsway E Kpene, Israel Bedzina, Bismark Mensah, Isaac K Agodey, Florence S Edziah, Richard V Duneeh

**Affiliations:** 1 Department of Medical Laboratory Sciences, School of Allied Health Sciences, University of Health and Allied Sciences, Ho, Ghana; 2 Department of Biomedical Sciences, School of Basic and Biomedical Sciences, University of Health and Allied Sciences, Ho, Ghana; 3 Reinbee Medical Laboratory and Wellness Centre, Ho, Ghana; 4 Salvation Army Hospital, Wiamoase-Ashanti, Ghana; 5 Agona Government Hospital, Agona-Ashanti, Ghana

**Keywords:** Hepatitis B Virus, Agona Government Hospital, Crude Prevalence, Infection, Year-on-year

## Abstract

**Objective:**

This study determined the burden and trend of Hepatitis B Virus (HBV) infection in individuals who visited Agona Government Hospital in Ghana.

**Design:**

A retrospective study.

**Setting:**

A single-centre study conducted at Agona Government Hospital.

**Participants:**

Clients who visited Agona Government Hospital.

**Main outcome measure:**

A hospital-based retrospective study was conducted. Data generated between January 2016 and December 2020 were extracted, cleaned, and validated using Microsoft Excel version 2019. Analysis was done using the Statistical Package for Social Sciences version 26.

**Results:**

A total of 2440 records from individuals who were tested for HBV were analysed. The records indicated varying numbers of tests each year, with 28.9% in 2016, 23.1% in 2017, 20.6% in 2018, 13.6% in 2019, and 13.7% in 2020. Most of the clients were aged 25-44 (46.3%). The majority of the clients were married (53.2%) and females (65.3%). The cumulative crude prevalence of HBV infection was 57.44 per 10,000. The prevalence of HBV varied across age categories and genders. A decline in prevalence was observed from 2016 to 2017, with a trough in 2018, followed by a rise through 2019 and peaking in 2020. The crude prevalence exhibited a peak in February and a trough in September, with inconsistent patterns throughout the year.

**Conclusion:**

The high burden of HBV underscores its significance as a pressing public health issue. There is therefore an urgent need for public health interventions to curb the spread of the infection.

**Funding:**

None declared

## Introduction

Hepatitis B Virus (HBV) causes a life-threatening infection of the liver. The disease is associated with different clinical outcomes. Viral hepatitis is a critical public health challenge in the entire continent of Africa and in about 80% of countries around the globe.^1^ The prevalence of chronic HBV among adults infected in adult life is 5% to 10%, and 85% to 90% of those infected in infancy. Every year, more than half a million HBV-related deaths are recorded globally.^2^ Because HBV infection is so widespread throughout the world and frequently causes chronic disease, cirrhosis, and liver cancer, it is a primary worldwide public health concern. The global incidence of chronic HBV varies from 0.1% to over 20%.^3^ The burden of the disease varies from one geographical region to another. In Europe and the Americas, approximately 1% of the population is chronically infected. The risk of being infected with HBV in one's lifetime in most countries in Africa and Asia, including parts of the Middle East, is estimated to be more than 60%. Understanding the epidemiological aspects of HBV infection is crucial for implementing evidence-based and cost-effective public health and clinical interventions at both national and international levels.^5^

The disease is endemic in sub-Saharan Africa and East Asia, affecting approximately 5–10% of the adult population.^6^ In Africa, there are estimated 65 million HBV carriers, who carry a 25% mortality risk. The percentage of people with HBV infection in sub-Saharan Africa varies between 9% and 20%. The epidemiological data used to calculate this high prevalence remain inadequate in the African region.^7^

There is no data on the prevalence of the hepatitis B Virus in the district where the study was conducted. However, the prevalence in Ghana ranges from 4.8% to 12.3% in the general population, from 10.8% to 12.7% in blood donors, and approximately 10.6% in pregnant women.^8^ This current study, therefore, focused on determining the burden and trend of HBV infection among individuals who visited Agona Government Hospital in the Sekyere South District of the Ashanti Region, Ghana.

## Methods

### Study design

This study was a hospital-based retrospective study conducted at the Agona Government Hospital in the Sekyere South District of the Ashanti Region. The study reviewed secondary data of individuals who visited the hospital and were tested for HBV between January 2016 and December 2020.

### Study site

The study was conducted at the Agona Government Hospital in the Sekyere South District of the Ashanti Region, Ghana. According to the 2010 Population and Housing Census, the population of Sekyere South District is 94,009. Females constitute 52.5 %, and the rest are males. Approximately 47% of the population resides in rural areas.^9^

### Inclusion and exclusion criteria

All records of HBV testing from January 2016 to December 2020 were included in the study. However, records with incomplete data (missing age, gender, and test results) were excluded from the study.

### Laboratory testing and quality control

The ABON Hepatitis B Surface Antigen Rapid Test kit was used to test for Hepatitis B Surface Antigen (HBsAg) in whole blood, serum, or plasma samples from participants. In conducting the test, two drops of serum or plasma (approximately 50 µL) were introduced into the sample well on the cassette and then allowed for 15 minutes for the appearance of coloured lines. A negative result was indicated by the presence of one coloured line in the control region (C) only.

A positive result was indicated by the presence of a coloured line in the control region (C) and test (T) line. The absence of the control line characterises an invalid result. In-house-generated known positive and known negative control sera were used as quality control materials. The sensitivity and specificity of the test kits were 99.8% and 99.2%, respectively.

### Data collection

Archived data were retrieved from the hospital's laboratory records using a pre-designed data-capturing instrument. The retrieved demographic parameters included age, gender, and marital status. Test results of the infectious marker of the virus were also retrieved for analysis.

### Data handling and analysis

Data was extracted, cleaned, and validated using Microsoft Excel version 2019. It was later exported to the Statistical Package for Social Sciences (SPSS) for statistical analysis. A descriptive analysis was done. Age and sex-stratified crude prevalence of HBV infection among the population of clients seeking healthcare at Agona Government Hospital was obtained using the Sekyere South District 2010 population and housing data of the Ghana Statistical Service. Population weights were calculated as the sum of people who were within an age-specific group (n) divided by the total population in the district (N). Year-on-year and month–on–month crude prevalence rates of HBV infection among clients seeking healthcare at Agona Government Hospital were also presented in graphs. This was calculated by dividing the number of cases per year or month by the total number of people per the respective year or month multiplied by 10,000 persons.

### Ethical considerations

Ethical clearance was obtained from the Research Ethics Committee (REC) of the University of Health and Allied Sciences, Ho, with protocol identification number UHAS-REC-A.11[51] 21-22. A written permission was also sought from the management of the Agona District Hospital, Ashanti Region. The confidentiality of participants' information and data obtained from the study was ensured by entering and analysing the data on a password-protected computer accessible only to the researchers.

## Results

A total of 2440 records of people who have been tested for Hepatitis B Surface Antigen (HBsAg) at the Agona Government Hospital, Sekyere South District, were retrieved from 2016 to 2020. Seven hundred and six, representing 28.9% of records, were obtained in 2016, 564 (23.1%) in 2017, 503 (20.6%) in 2018, 332 (13.6%) in 2019, and 335 (13.7%) in 2020.

Out of the 2440 records of the study participants retrieved, most (1130/2440, 46.3%) of them were within the age category of 25-44, followed by 1028 (42.1%) within the age category of 5-24 and the least 27 (1.1%) within the age category of <5. Within the age categories of <5, 45-64, and >64, the highest distribution of study participants was as follows: 13 (48.1%) in 2018, 48 (25.0%) in 2020, and 14 (22.2%) in both 2016 and 2018, respectively. In 2016, most of the participants, 305 (29.7%), fell within the age category of 5-24, while 346 (30.6%) were in the 25-44 age category. Generally, the majority of participants were married (1297, 53.2%) and female (Table 1).

**Table 1:** Sociodemographic characteristics of study participants

Parameter	Total	2016	2017	2018	2019	2020
	N (%)	N (%)	N (%)	N (%)	N (%)	N (%)
**Total**	2440(100.0)	706(28.9)	564(23.1)	503(20.6)	332(13.6)	335(13.7)
Age*	26.0±6.0	26.0±6.0	26.0±6.0	27.0±6.0	24.0±7.0	28.0±9.0
**Age Category (years)**						
**<5**	27(1.1)	4(14.8)	3(11.1)	13(48.1)	5(18.5)	2(7.4)
**5 - 24**	1028(42.1)	305(29.7)	245(23.8)	194(18.9)	163(15.9)	121(11.8)
**25 - 44**	1130(46.3)	346(30.6)	276(24.4)	241(21.3)	114(10.1)	153(13.5)
**45 - 64**	192(7.9)	37(19.3)	28(14.6)	41(21.4)	38(19.8)	48(25.0)
**≥65**	63(2.6)	14(22.2)	12(19.0)	14(22.2)	12(19.0)	11(17.5)
**Gender**						
**Female**	1594(65.3)	477(29.9)	419(26.3)	343(21.5)	181(11.4)	174(10.9)
**Male**	846(34.7)	229(27.1)	145(17.1)	160(18.9)	151(17.8)	161(19.0)
**Marital Status**						
**Single**	981(40.2)	277(28.2)	234(23.9)	196(20.0)	160(16.3)	114(11.6)
**Married**	1297(53.2)	389(30.0)	292(22.5)	282(21.7)	144(11.1)	190(14.6)
**Divorced**	8(0.3)	0(0.0)	3(37.5)	5(62.5)	0(0.0)	0(0.0)
**Widowed**	154(6.3)	40(26.0)	35(22.7)	20(13.0)	28(18.2)	31(20.1)

Data are presented as figures and percentages in parentheses.

* is presented as median ± Median Absolute Deviation

Within the five years under review, the cumulative crude prevalence of HBV infection stood at 57.44 per 10, 000. HBsAg cases (females, males) across the various age categories were 2, 2; 121, 79; 163, 109; 33, 21 for <5, 5 – 24, 25-44, and 45 - 64, respectively. The highest crude prevalence of HBsAg was recorded among females (143.74 per 10000) who were between 25-44 years, followed by age category 5-24 years (54.45 per 10000), and the least prevalence of 3.28 per 10000 was recorded among the age category of <5 years. The same pattern was observed with the age-standardised prevalence/10,000. Prevalence of HBsAg cases among females was 30.99/10000, 25.83/10000, and least (0.44/10000) for age categories 25-44, 5-24 and <5 years, respectively. The same pattern of the crude prevalence/10,000 and the age-standardised prevalence/10,000 of HBsAg cases for females was also observed for males, with both the highest and least cases recorded within the age category; 25-44 and <5, respectively, as shown in Table 2.

**Table 2:** Age- and gender-stratified crude prevalence of HBsAg infection among the population of participants seeking healthcare at Agona Government Hospital

Parameter	Population		HbsAg Cases		Crude prevalence/10,000			Age-Standardised Prevalence/10,000	
	Female	Male	Female	Male	Female	Male	P weight	Female	Male
**<5**	6102	6475	2	2	3.28	3.09	0.13	0.44	0.41
**5 - 24**	22221	22372	121	79	54.45	35.31	0.47	25.83	16.75
**25 - 44**	11340	8931	163	109	143.74	122.05	0.22	30.99	26.32
**45 - 64**	6300	4817	33	21	52.38	43.60	0.12	6.19	5.16
**≥65**	3355	2096	7	3	20.86	14.31	0.06	1.21	0.83
**Total**	**49318**	**44691**	**326**	**214**	**274.71**	**218.36**	**1.00**	**64.67**	**49.47**

**Figure 1:**
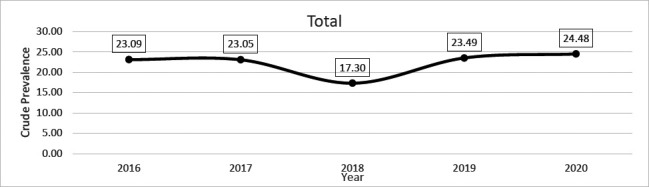
Year-on-year crude prevalent rates of HBsAg infection among the population of patients seeking healthcare at Agona Government Hospital

Population figures and weight were obtained using the 2010 population figures for the district. (Ghana statistical service, 2010). P weight, population weight. In Figure 1, a slight decline was observed in the crude prevalence of HBsAg cases between 2016 and 2017, with a trough in 2018. A quite opposite phenomenon is observed from 2018 to 2020, as there was a gentle rise through 2019 and peaking in 2020.

**Figure 2:**
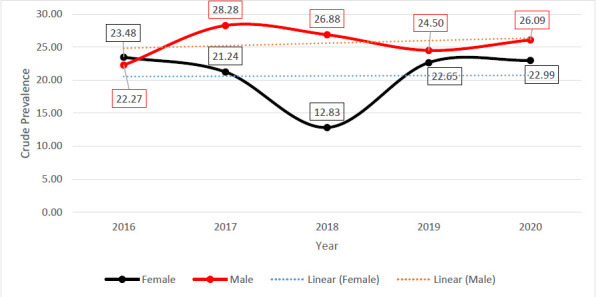
Year-on-year crude prevalent rates of HBsAg infection by gender among the population of individuals visiting the Agona Government Hospital

Generally, there was a gradual increase in male cases over the period, while females experienced a relatively stable case rate over the same period. The crude prevalence of HBsAg cases per 10,000 population for females, stratified by year, peaked at 23.48 in 2016, declined gently to 21.24 in 2017, and further to its trough (12.83) in 2018. However, the prevalent rates between 2018 and 2020 bucked the trend. Concerning the male crude prevalence of HBsAg cases per 10000 population, there was a steep rise from 2016 (23.48) to 2017 (28.28). This was followed by a gentle decline to 2019 (24.50) and finally a gentle rise to 26.09 in 2020 (Figure 2).

**Figure 3:**
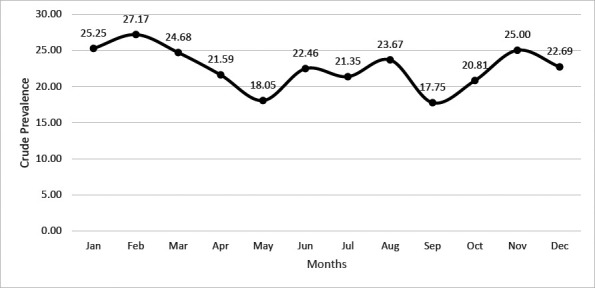
Total month–on–month Crude prevalent rates for the study participants

An inconsistent rise and decline pattern was observed in the crude prevalent rate of HBsAg cases per 10000 population for the study participants from January to December. The trough and peak were observed in September and February, respectively, as indicated in Figure 3.

**Figure 4:**
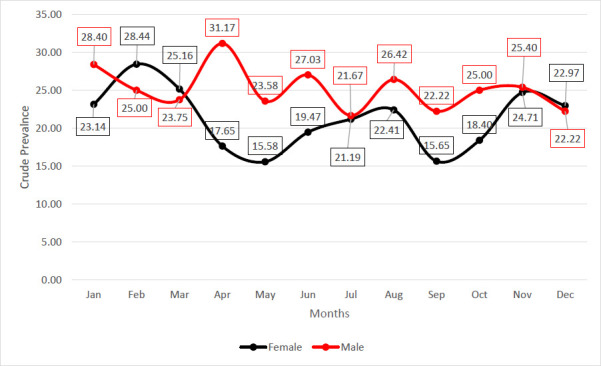
Month–on–month crude prevalent rates of HBsAg among the study participants stratified by gender

As shown in Figure 4, the peaks and troughs in the prevalence of HBsAg cases per 10,000 population for males and females were observed in April and February, respectively, with an inconsistent rise and fall in July and May.

## Discussion

Hepatitis B is an infectious virus that affects the liver and can be contracted through exposure to the blood or other bodily fluids such as wound discharge, vaginal fluids and semen of an infected individual.

Despite the existence of effective vaccines and treatment strategies, the disease continues to pose a significant global health challenge, potentially resulting in acute and chronic conditions, severe liver failure, and even malignancy.^10^ The current study analysed a total of 2440 records of individuals tested for Hepatitis B Surface antigen (HBsAg) at the Agona Government Hospital in the Sekyere South District over the period from 2016 to 2020.

The results of the study indicate a cumulative crude prevalence of Hepatitis B Virus (HBV) infection over the five years at 57.44 per 10,000. The prevalence of HBsAg cases, distinguishing between females and males across different age categories, is presented. The number of cases per 10,000 for females and males is as follows: <5 years (2, 2), 5 – 24 years (121, 79), 25-44 years (163, 109), and 45 - 64 years (33, 21). Notably, the highest crude prevalence of HBsAg was observed among females, particularly those aged 25-44, with a rate of 143.74 per 10,000. This was followed by the age category of 5-24 years with a prevalence of 54.45 per 10,000, while the lowest prevalence of 3.28 per 10,000 was recorded among those under 5 years old. A review by Ofori-Asenso, Agyeman^6^ Identified the prevalence of HBV in Ghana, as detected by HBsAg seropositivity, to be high. Another study among blood donors by Osei *et al.*^11^ reported HBV prevalence among the 30–39 year age group. In this study, individuals under five years old recorded the lowest prevalence of HBV infection. This could be due to the introduction of the Pentavalent vaccine at week six of life and the promotion of childhood immunisation. This has led to the decline in vaccine-preventable diseases in Ghana^12^, including the HBV. The Pentavalent vaccine includes vaccines against diseases such as measles, polio, tuberculosis, hepatitis B, and yellow fever^13^-^15^. The Ghana Health Service is responsible for implementing the national immunisation programme, which aims to provide vaccines to all eligible persons, as it is a vital component of the country's public health strategy against under-five morbidity and mortality^13^. The pattern of prevalence persists when considering age-standardised rates. Among females, the age-standardised prevalence of HBsAg cases per 10,000 was 30.99, 25.83, and 0.44 for the age categories 25-44, 5-24, and <5 years, respectively. Interestingly, the same pattern is observed among males, with the highest and lowest cases recorded within the age categories 25-44 and <5, respectively.

The results underscore the consistency in the prevalence patterns for both crude and age-standardised rates of HBsAg cases among females and males. The findings suggest a higher vulnerability to HBV infection among females, particularly those in the 25-44 age group, while the lowest prevalence is seen in the age group under 5 years. The high HBV infection rates among individuals aged 25-44 could be attributed to increased sexual activity in this age group, which increases the risk of transmission through unprotected sex^16^,^17^, lack of vaccination or incomplete vaccination series, which leaves individuals susceptible to infection^18^,^19^, and engagement in high-risk behaviours such as tattoos, piercings, or sharing personal care items.^20^,^21^ The high prevalence of the infection in this age group among the study population could lead to liver cirrhosis and hepatocellular cancer.^22^,^23^ Therefore, screening should be targeted at this age group and treatment offered to those who require it, and vaccination for those who test negative. This will help reduce the rate of morbidity and mortality of HBV in Ghana.

Generally, females are more likely to be HBsAg positive than males.^11^ In contrast, the prevalence of infected male individuals with HBV was higher than that of infected female individuals in a study by Kolou *et al.* 7 Our study findings are consistent with a study by Al-Mendalawi^24^, where participants within the 21–40 year age group had the highest age-based HbsAg prevalence. The high prevalence in this age group may be attributed to the sexually active nature of this group, illicit drug use, tattooing, and body piercing which are risk factors associated with HBV transmission and are prevalent among this age group.^24^ This finding is further supported by Kolou *et al.*^7^

This study's findings provide valuable insights for public health interventions, emphasizing the need for targeted efforts, such as vaccination campaigns and awareness programs, to address the specific demographic groups with higher prevalence rates. Additionally, monitoring and preventive measures for males, mirroring the patterns observed in females, could contribute to a comprehensive strategy in managing and reducing HBV infection rates within the population.

This study is limited because it is a single-centre study, and its findings cannot be generalized. The study focused solely on reporting the prevalence of hepatitis B without providing a comparison to the prevalence in different subgroups, such as the general population, immunocompromised patients, pregnant women, and blood donors. This lack of comparative data makes it challenging to assess the significance of the reported prevalence within these specific populations. The absence of such comparative information limits the generalizability and applicability of the findings, as the study's results may not accurately reflect the actual burden of hepatitis B across various population segments.

## Conclusion

The burden of Hepatitis B infection underscores its significance as a pressing public health issue. Given the high burden of the disease, there is an urgent need for public health interventions and strategic policy directions to control the spread and prevent a potential future outbreak. It is recommended to regularly screen the general population for both viral infections, aiming to improve early diagnosis, confirm eligibility for treatment, deliver timely treatment, and educate and raise awareness about preventive measures.
